# Statistical Report of the Sickness, Mortality, and Invaliding among the Troops in the West Indies

**Published:** 1840-01

**Authors:** 


### ART. III.

1. *Statistical Report of the Sickness, Mortality, and Invaliding among the Troops in the West Indies; prepared from the Records of the Army Medical Department and War-office Returns.* Presented to both Houses of Parliament by command of her Majesty.—*London*, 1838. pp. 103, with a Supplement of 40 pp.

2. *Statistical Report of the Sickness, Mortality, and Invaliding among the Troops in the United Kingdom, the Mediterranean, and British America; prepared from the Records of the Army Medical Department and War-office Returns.* Presented to both Houses of Parliament by command of her Majesty.—*London*, 1839. pp. 172.

WE have already recorded our sense of the value of these documents, and presented our readers with a view of a portion of their contents, calculated, we are convinced, to justify the opinion we expressed. Want of space compelled us to limit our extracts and remarks in the former Number mainly to the statistics of two classes of the diseases, which are examined numerically in these volumes, fevers of the tropics, and pulmonary affections. These were selected at once for their paramount importance and the great value of the information produced regarding them; a value marked, we admit, more frequently by the subversion of former than the perfect establishment of new opinions: but we were well pleased to have advanced so far, regarding, as we must, the destruction of error as the first step, and this an essential one, in the advancement of truth. There are, however, other and very important matters in these volumes, treated with the same preciseness as to facts, and the same sobriety of deduction which characterized the portions we have already noticed; and of these we shall present a concise, but, we trust, comprehensive view to the reader.

1. *Diseases of the Liver.* On this subject, Major Tulloch makes the following remark, when treating of the maladies and mortality of the Windward and Leeward Island command in the West Indies: "Though this class of diseases is by no means so common as in the tropical regions of the eastern hemisphere, it is nearly thrice as prevalent as among troops in the United Kingdom, and occasions about five times as high a rate of mortality." On tracing the prevalence and mortality of this disease through the different West Indian commands, we find in that of the Windward and Leeward Islands there are admitted 1,946, of whom die 161, or one in twelve; and when we take the proportion to the mean strength, it is stated that 22 per 1000 are admitted, and 1·8 per thousand die. There are, in the different stations throughout this command, considerable varieties in the proportions in which the troops there have contributed to produce this general ratio. At Trinidad, for example, the proportion of mortality per mean strength from this disease is 1·1, in Tobago 2, and in Grenada 4·5 per thousand. This last very striking excess above the general ratio, the reporter acknowledges himself to be utterly unable to explain. The proportions stated refer to the white troops only. The blacks suffer from this class of diseases and from all those which principally produce the mortality in a much smaller proportion than the whites, if we except pulmonary diseases and eruptive fevers, to which classes of disorders they manifest a much greater proclivity than Europeans.

Continuing still to trace the mortality from diseases of the liver through the different commands, we find that in Jamaica, notwithstanding its high temperature, this is only 1 for 1000; and that, as compared with Britain, the ratio of admissions is as 10 to 8, and the deaths as 1 to $\frac{4}{10}$. Two of the stations, Fort Augusta and Spanish Town, present examples of striking exemption from these diseases, the mortality from this source being only 0·3 per 1000, or exactly the same as in civil life in England. We can discover nothing in these stations to account for this comparative exemption. Their temperature is not below the average of the island, in which the extremes in stations occupied by our troops are as follows: at Kingston, average maximum 85°, minimum 76½°; at Maroon Town, average maximum 80½°, minimum 68½°. At Fort Augusta, under the very walls of the fort, is an extensive marsh or lagoon, interspersed with small islands, covered with mangrove bushes, and abounding in every species of decayed vegetation, from which issue most offensive effluvia. In Spanish Town, the temperature during the day is much higher than at the other stations; and the soil being clayey and tenacious, partial swamps are produced by heavy rains. "The country, as far as the foot of the mountains, being a dead level, and no artificial means employed to carry off the superabundant moisture, it remains until evaporated by the sun's rays; and when the land winds blow over the ground thus saturated, they are supposed to have considerable influence in the production of fever." There can be no doubt of the abundance of rum and the facility of procuring it in both these situations. We thus observe the reputed causes of disease of the liver; heat, marshy effluvia, and ardent spirits, existing in abundance, whilst the disease prevails to a very insignificant extent, without any assignable reason for the exception, but the vague and conjectural one, of some specific power in the climate counteracting their operation.

In the other West Indian commands, the ratio is deduced from so small a number of cases, as can hardly be considered to constitute a fair ground of reasoning: we consequently pass on to the numbers stated in Great Britain, the Mediterranean, and North American stations. In our own country, we find that the deaths from liver disease, in civil life, amount (the ratio being taken from subjects insured in the Equitable Office from 1801 to 1832,) to ·3 per 1000, and among the troops to ·4 per 1000. The mortality from this class of diseases is exceedingly low in certain descriptions of force, amounting* to 1 in 10,000 annually in the foot guards, amongst whom we had occasion, in our former article, to point out the extraordinary prevalence of consumption; whilst the returns from the West Indian depôts show the ratio there to be the same. In the dragoon guards and dragoons, 4 in 10,000 die annually of liver disease; and in the household cavalry, 5 in the same number. In passing to the Mediterranean stations, we find the mortality in Gibraltar, 4 per 10,000 annually, or the same as among troops in the United Kingdom; at Malta, 11 in 10,000, or nearly three times as high as in the former station; whilst among the civil population of this latter garrison, it is extremely low, being only 11 in 100,000, or one tenth of the proportion among the troops. A supposition of Major Tulloch's may serve at once to explain the great difference between the prevalence of these diseases in two stations, not materially differing from each other in climate, Gibraltar and Malta, and the still more striking discrepancy between its effect on the military and civil population in the latter station. He remarks that the troops in Malta may have contracted the disease during a previous course of service in climates where it was very prevalent. We think this very probable; but, however it may be, the fact of the great exemption of the Maltese is sufficient evidence that heat, for many months, little inferior to that of tropical regions, is inadequate to produce a prevalence of liver disease. In the Ionian islands, the rate of mortality from this malady is only 8 in 10,000, and this low annual rate pervades all the islands, Zante alone excepted, where 20 in 10,000 die yearly—a higher rate than exists in any other Mediterranean station: no explanation can be found of this discrepancy.

In British America, this disease contributes but little to the average mortality among the troops. In the Bermudas, 5 in 10,000 die of it

* For the sake of greater perspicuity, we state the proportion in integers.

annually, and in Nova Scotia and New Brunswick, only 2 in the same number; whilst in Canada, the admissions and deaths are in the same low proportion as in these latter colonies. Major Tulloch here makes the following remark, which is equally applicable to Canada, New Brunswick, and Nova Scotia:

"The prevalence of this class of diseases is almost the same as among the dragoon guards and dragoons serving in the United Kingdom, and the mortality is only half as high. This fact tends to throw very considerable doubt on the supposed influence of spirituous liquors in inducing affections of the liver, at least in a cold climate; for owing to the low price of these in Nova Scotia and New Brunswick, there are few stations where intemperance is carried to a greater extent; yet not only do the troops suffer less from diseases of the liver than at home, but the proportion of deaths is only two thirds as high as among persons insured in the Equitable office, who, from their rank in life, as well as the caution exercised in their selection, are by no means likely to be addicted to that vice." (*British-American Report*, p. 17, *b.*)

On surveying the returns of affections of the liver, we have certainly been impressed with the scantiness of their contributions to the general mortality of most of the districts where their influence has been traced; and it will occur to the reader, either that such affections figure much too highly in the opinion and language of certain medical men in this country, or that they are the most curable of human maladies. These returns, however, disprove the latter branch of the alternative, for we find the proportions of deaths to the number treated by no means insignificant. The fact appears to be, that throughout the western hemisphere, diseases of the liver are very rare in comparison of those of the lungs and of the stomach and bowels; and though in some localities they are more frequent than diseases of the brain, they contribute much less than these do to the amount of mortality. We regret that we have no East Indian returns from which to deduce a comparison regarding the prevalence of liver-diseases between the western and eastern hemispheres.

2. *Diseases of the Stomach and Bowels.* In the West Indian Report we find that in the Windward and Leeward command, 20·7 per thousand die annually of these complaints, the proportion attacked being 421 per thousand, of which one in twenty dies; whereas, in this country, the admissions for such diseases are only 95 per 1000, and the cases are so mild that only one proves fatal for 2000 of the mean strength. Dysentery and diarrhœa are the great sources both of admissions and mortality from this class in the West Indies. Acute dysentery is directly fatal to 1 in 26 of those attacked, and a very great mortality results from a considerable proportion of the cases passing into the chronic form, of which cases 1 in 5 is fatal. Diarrhœa is a very frequent disease, and is fatal to 1 in 42 of those it attacks.

In the Jamaica command, the annual admissions from this class of diseases amount to 238, and the deaths to 5·1 per thousand of the mean strength, the admissions being only about one half, and the deaths scarcely one fourth part as high as in the Windward and Leeward command. The diseases, dysentery and diarrhœa, which contribute most largely to the sickness and mortality, are less prevalent and much less fatal in Jamaica; for, whilst the admissions from the whole class of bowel-complaints amount only to 238 per 1000, acute dysentery is fatal to 1 only in 40, and diarrhœa to only 1 in 123 of those attacked. The only discernible reason for this difference between the two commands is, that in Jamaica the troops have twice as much fresh provisions as those in the Windward and Leeward command, "which," as Major Tulloch justly remarks, "if it does not prevent the occurrence of the disease, may obviously have a very material effect in modifying its virulence, by supplying a more digestible and less irritating diet than salt meat."

Passing over the other two West Indian commands, as containing too small a number of troops to furnish *data* for a fair inference, we proceed to trace the prevalence of diseases of the stomach and bowels among troops in the United Kingdom. In the dragoon guards and dragoons, the annual admissions amount to 94 per 1000, and the deaths to 8 per 10,000 of the mean strength, a very small mortality, amounting to 1 only in 117 of those attacked; whilst in other descriptions of force, the proportionate mortality is still lower, being in the foot guards 7, in the household cavalry 3, and in the West Indian depôts 4 in 10,000.

When we turn to Gibraltar, we find bowel complaints considerably more prevalent and fatal than in the United Kingdom, the admissions being 186, and the deaths 2·1 in 1000 of the mean strength annually. The mortality mainly arises, as in the West Indies, from acute and chronic dysentery and diarrhœa; but, although these are the most prevalent and fatal diseases, they are so in a much less degree than in the West Indies, whilst the pure inflammations of the intestinal canal are of nearly equal prevalence and fatality in both these colonies and in Great Britain. The reporter refers to the returns from the civil hospital of Gibraltar, and finds that bowel complaints do not there bear so high a proportion to other diseases as among the troops.

"This is the only way in which we can make the comparison, and though it does not supply such accurate grounds for our deductions as could be wished, it certainly adds to the presumption that the tendency to diseases of the bowels at this station may be increased by the large proportion of salt meat issued to the troops, especially as the officers suffer so little from them, and there is nothing in the duty of the troops likely to have created such a peculiarity. Whatever may be the opinion in regard to the possibility of this diet creating such diseases, there seems little doubt that for those who may have been suffering under them, and have recently come out of hospital, it is most inappropriate, as having a greater tendency to induce a relapse than the milder and more digestible nutriment afforded by fresh provisions."(*Mediterranean Report*, 13, *a.*)

A confirmation of these views is derived from a comparison drawn by Major Tulloch, between the mortality arising from these complaints among the troops, and the civil population of Malta. Bowel complaints are more fatal among the troops in this island than in Gibraltar, the mortality being at the annual rate of 3·6 per thousand of the mean strength, whilst the admissions are only 155 per 1000, or considerably lower than in the latter garrison. An extract from a return of the deaths among the civil population of the island, for thirteen years, shows an extreme accordance between the troops and the inhabitants in the mortality resulting from these diseases, the deaths in the latter class being 3·8 per 1,000, or a small fraction higher than among the soldiers. The diet of the troops is good and ample, and their meat is fresh.

In the Ionian islands, the admissions from these complaints are 156, and the deaths 3·5 per 1000, being in the same proportions as in Malta; and this latter ratio is very equally preserved throughout the islands, with the exception of Santa Maura, where the mortality from this cause is considerably below the average, the deaths from bowel complaints being only 2 per 1000, whilst those from fever are more than double what takes place in any other island. This tends to confirm views much insisted upon by writers on the influence of malaria in tropical regions, that bowel complaints are there equally with fever the products of malaria; that, according to certain varieties in its intensity or composition, the one disease or the other is produced; and that, consequently, a great prevalence of the one disease necessarily excludes that of the other. Whatever may be thought of the more theoretical parts of this opinion, there can be no doubt that the occurrences at Santa Maura are in confirmation of the plain fact on which they are founded.

When we transfer our attention to British America, we find that in Bermuda, the admissions, on account of these diseases, amount to 415, and the deaths to 5·3 per thousand, the former ratio being as high as in the Windward and Leeward command in the West Indies, though the mortality is much smaller. In explanation of the number of admissions, it should be observed, that Bermuda is in latitude 32° north, and has a high temperature, its summers especially being very hot; and, besides, there being much difficulty in procuring a sufficiency of fresh meat in a group of very small islands, salt rations are much employed, and the water, moreover, is brackish. In the colder latitudes of North America, bowel complaints are much less prevalent and fatal. In Nova Scotia and New Brunswick, for instance, the admissions are only 94 and the deaths 1·5 per 1000; and when from this latter ratio we deduct the deaths in the Fifteenth Foot, which brought with it, from the West Indies, several invalids labouring under chronic affections of the bowels, the proportions of both the admissions and deaths are substantially the same as in the United Kingdom. So in Upper and Lower Canada, the admissions are 155 and the deaths 1·3 per 1000; but making suitable deductions, on account of the mortality in the Second Battalion of the Sixtieth, which brought with it, from the West Indies, many soldiers worn out by dysentery, we find the deaths in nearly the same proportion as in Nova Scotia, New Brunswick, and the United Kingdom.

These statistics confirm the opinion generally entertained, that bowel complaints are especially prevalent and fatal in warm climates, whilst in the colder latitudes they are comparatively unfrequent and very mild. They afford ample evidence too, that irritating and indigestible diet, particularly salt meat, cooperates very powerfully with the climate in producing these diseases. In the details of the returns, there is a fact manifested, which is calculated to impress very forcibly on any individual accustomed to observe diseases at home only, the extreme prevalence and fatality of diarrhœa in warm climates, especially where irritating diet is conjoined with the influence of climate. The accuracy of the military reports on this point receives confirmation from some statistical details of the Lisbon hospitals, which we recently examined,—Diarrhœa contributed in a surprising degree to the sickness and mortality in these institutions. Now we happen to know that the food of that class in

Lisbon, who are likely to be inmates of a public hospital, consists mainly, in addition to fruit and vegetables, of salt fish.

3. *Diseases of the Brain.* The statistics of this class of diseases furnish much interesting information. In the Windward and Leeward command in the West Indies, the admissions are 28, and the deaths 3·7 per 1000; and deducting the cases of *delirium tremens*, to which more than one half of the sickness and mortality from this class of diseases are owing, the admissions are 12, and the deaths 1·6 per 1000, or just double of what takes place from the same cause in the United Kingdom. In Jamaica the admissions are only 14, and the deaths 2·6 per 1000, the cases of *delirium tremens* being only one third as numerous as in the Windward and Leeward command. In the United Kingdom the admissions are 6 in 1000, and the deaths 8 in 10,000, or only half of what occurs in civil life, according to the tables in the Equitable office. In explanation of this very striking discrepancy, Major Tulloch justly remarks that this class of diseases is supposed to be a greater source of mortality in the upper walks of life, from which the subjects of the Equitable office are drawn, than among the lower orders, whence our soldiers are recruited.

In Gibraltar the admissions are 6 in 1000, or the same as in the United Kingdom, whilst the deaths are still fewer, amounting to only 5 in 10,000. In Malta the admissions and deaths are precisely in the same proportions as in the United Kingdom: viz. the former 6 in 1000, the latter 8 in 10,000. In the Ionian islands, the admissions are 10, and the deaths 1 per 1000. When we again turn to the Western hemisphere, we find in Bermuda the admissions 17 and the deaths 2 per 1000; in Nova Scotia and New Brunswick, admissions 11 and deaths 1·3; and in Canada, admissions 14 and the deaths 1·2.

From these proportions, we perceive that affections of the brain are generally more prevalent in warm than cold climates, but that the prevalence or paucity is by no means in proportion to the warmth or coldness of the climate. This will be observed if we compare Gibraltar and Malta with Bermuda, and the United Kingdom with Nova Scotia, New Brunswick, and the Canadas: in fact, we perceive throughout a predominance of brain affection in the western compared with the eastern hemisphere. It would be interesting to discover whether this preponderance is in diseases of the brain in general or in some special affection. The following facts evince that the difference is mainly owing to the extreme prevalence and fatality of *delirium tremens* among the troops in our American colonies. In the Windward and Leeward command, *delirium tremens* gives rise to four sevenths of the admissions from all brain diseases, and to the same proportion of the mortality from them. In Jamaica more than one fourth of the admissions and nearly one third of the deaths arise from the same cause. In Bermuda, more than one half of the admissions and nearly one half of the deaths are attributable to it; in Nova Scotia and New Brunswick half of the cases and one fourth of the deaths; and in the Canadas, three eighths of the cases and one fourth of the deaths. When we turn to the European reports we find in Gibraltar, that of brain diseases, little more than one ninth of the admissions, and less than one sixth of the deaths, are from *delirium tremens*; in Malta, less than one sixth of the admissions, and exactly one sixth of the deaths; and in the Ionian islands, one fourth of the admissions and three sevenths of the deaths, being the largest proportions in any European colony.

There can be no doubt that the great mortality from this disease in our American possessions arises from the facility of indulgence in ardent spirits which they afford. In our European colonies, the facilities of indulgence in liquor are considerable, and there can be little doubt of our troops availing themselves of them, but the effect is less pernicious, probably from wine being the beverage generally employed, which, however injurious when taken in excess, is much less prone to produce the brain fever of drunkards than ardent spirits. How many lives might be saved, could the unfortunate tendency of the British soldier to drunkenness be corrected, may be conceived, when we consider that where the ratio of *delirium tremens* to other affections of the brain is the highest, there do these last exist in the greatest abundance and severity; so that these reports show that habits of intoxication are the most fertile source of affections of the brain in general. Regarding the influence of mere heat on their production, Major Tulloch is of opinion that it is not very great (*West Indian Report*, p. 9); but on the conjoint influence of high temperature and habits of intoxication, he makes the following striking remark:

"The proportion admitted at this station (Gibraltar) is exactly the same as among the dragoon guards and dragoons at home, and the ratio of deaths is even lower; it may be necessary to state, however, that in order to obviate the consequence of exposure to solar influence, a precaution is adopted in this command of confining the troops to barracks during summer from nine or ten o'clock in the morning till four or five in the afternoon. Cases of *delirium tremens* are comparatively rare, *not more than half as many have died by it in the course of nineteen years, as in the Mauritius during one year alone out of half the strength.* Though intemperance is by no means uncommon among the garrison, it seems neither to be carried to that extent nor to be productive of the same prejudicial effects, morally or physically, as in tropical climates, where consequently there is the more urgent necessity for restrictive measures to prevent an indulgence so pernicious to the health and discipline of the soldier." (*Mediterranean Report*, p. 15, *a.*)

4. *Diseases of the Heart.* These are not sufficiently frequent among soldiers to receive specific mention in the very luminous abstracts of the returns given by Major Tulloch; but we find them in the original returns, under various designations, some of them distinct, others sufficiently vague. We are impressed with their paucity, especially in comparison with the disease whence they so often in civil life derive their origin, rheumatism. Thus we find in the West Indies, that in the Windward and Leeward command there are 4202 cases of rheumatism and 11 of carditis, and 23 returned under the vague head of palpitatio. In Jamaica there are 1479 cases of rheumatism and 8 of carditis. In the United Kingdom there are 2244 examples of rheumatism, whilst the dragoon guards and dragoons furnish 24 cases, named carditis, the foot guards 5 cases of death from *morbus cordis*, and 2 from carditis; and the household cavalry one death from pericarditis, the deaths only, not the admissions, from these diseases being specified in the returns of the household troops. In Gibraltar, there are 2309 cases of rheumatism, 12 of carditis, and 8 of *morbus cordis;* in Malta, 1383 cases of rheumatism and 4 of carditis; and in the Ionian islands, 2428 cases of rheumatism, 4 of carditis, and 20 of *morbus cordis.* In our North American possessions, there are in Bermuda 390 cases of rheumatism and one of carditis; in Nova Scotia and New Brunswick, 1310 cases of rheumatism and 6 of carditis; and in the Canadas, 2427 of rheumatism and 11 of carditis.

We have no means of proving it from the statistics of civil life, but we are convinced by these returns that disease of the heart is less frequent and bears a lower proportion to rheumatism among British troops than among the labouring class in this country from whom they are recruited. This admits a ready explanation in the greater care taken of sick soldiers, and the less haste in their return to active employment on the abatement of disease, than occur to persons of the same rank of society in civil life. This comparative paucity, however, of cardiac affection, and the great amount of rheumatism which these returns indicate, ought, we think, to convince M. Bouillaud that carditis is not an essential part of rheumatism—not the rule, as he terms it, but the exception, or we should find, notwithstanding every allowance for the very advantageous circumstances in which soldiers are placed in respect to medical care and treatment, the latter disease laying the foundation of the former more frequently than it does.

5. *Epidemic Diseases.*—A. *Yellow Fever.* The reports contain much valuable information regarding epidemics, and as these occasional visitations, besides their intrinsic importance, present many interesting points of comparison with the ordinary diseases of any given climate, we shall transfer a portion of it to our pages. The facts mentioned regarding the yellow fever of Gibraltar, though detailed with the utmost brevity and with a scrupulous abstinence from all intermixture of speculative opinion, appear to us admirably calculated, not to *settle* the perplexed question of the origin of epidemics, but to render the adjustment of the controversy, which has long raged respecting it, more probable, by presenting distinct facts at a period when the minds of men are disposed to contemplate them with more equanimity than when the feelings are excited by the recent occurrence of such a visitation, and the disquisitions to which it gives rise.

Whilst the admissions from all the ordinary fevers of Gibraltar amount in nineteen years to 8165, and the deaths to 140, being a mortality of one in fifty-eight, the admissions from the epidemic fever of 1828 amounted to 1522, and the deaths to 423, constituting a mortality of one in three two-thirds. The strength of the garrison was 3494, of whom nearly one half were attacked by the disease, and one eighth perished. Now, in ordinary years, so far from the garrison of Gibraltar having suffered in any remarkable degree from fever, the proportion of deaths is only about one half higher, and the admissions about double what occurs from the same cause in this country.

Major Tulloch gives a summary of the preceding epidemics of yellow fever in this garrison. In that of 1804, there perished 54 officers, 864 soldiers, 164 soldiers' wives and children, and 4864 civilians. In 1813 it destroyed 461 of the troops and 883 inhabitants; and in the following year it again broke out, and 114 of the former and 132 of the latter class perished. From this time till 1828 there was no epidemic.

In many respects there was an accordance among the various epidemics, that of 1828 included. Though in certain of them a few scattered cases may have appeared earlier, yet in the latter end of August or the beginning of September was the commencement of the marked epidemic prevalence; the mortality attained its *maximum* about the middle of October, and, though no marked diminution either in prevalence or severity could be distinctly traced to the occasional reductions of temperature, which took place during the continuance of the disease, yet these epidemics have never been known to make their appearance at this station during winter, and they have always declined in severity as this season approached. Of all the means resorted to for the purpose of checking the progress of the disease, removal to an encampment beyond the precincts of the garrison has been most invariably successful. The place selected for this purpose was the neutral ground, and "though it is composed of materials which are supposed highly favorable to the formation of malaria," yet the epidemic has uniformly been checked in bodies of persons, civil or military, who have been removed thither; indeed it is stated by the surgeon of the Twelfth Regiment, that of 92 women and 190 children encamped in the neutral ground, during the whole period the epidemic of 1828 prevailed, not one was attacked, though in constant communication with the soldiers of the corps, who went daily from that encampment to do duty in the town, and of whom many were attacked with the disease on their return. Major Tulloch's account of the geological formation of Gibraltar is not such as would lead to the supposition that it would prove unhealthy; neither is it ordinarily so.

"The rock of Gibraltar is principally composed of gray limestone. The upper part is almost entirely devoid of soil, except in the gullies, and a few spots where it has accumulated by the action of the rains. The level piece of ground on which the principal streets of the town are built is composed of red sand, and towards the south side there is some light fertile mould, but it is exceedingly scanty; every spot available for the purpose, is laid out in garden-grounds, and wherever an adequate supply of soil and moisture can be obtained, the produce is most abundant. About 200 acres of that portion of the isthmus, termed Neutral Ground, have also been brought under cultivation, and furnish an ample supply of vegetables for the garrison. The whole surface of the rock, particularly on the western side above the town, is much intersected by deep gullies, in which, during winter, water occasionally lodges; they are, however, always dry in summer. To the southward are several extensive tanks, containing nearly two million gallons of water, for the use of the troops and shipping; but in no part is there any ground which can be designated as marshy, and in general it is necessary to dig to the depth of thirty or forty feet before water can be procured." (*Mediterranean Report*, p. 3, *a.*)

It is more reasonable to look for the causes of an extraordinary degree of sickness in some unwonted state of the seasons, than in a geological condition, which is permanent. Investigations in this direction, however, leave the subject as obscure as before. "There was nothing remarkable," says the reporter, "in the atmospherical phenomena which preceded the last epidemic: the summer was cool and pleasant, and the easterly winds so much complained of at the station, were more rare than usual; there was rather less rain, and fewer rainy days than had occurred during the two preceding years, but a smaller quantity had often fallen in other years, which proved remarkably healthy." Tables are given of the temperature, the barometric pressure, and the quantity of rain during a healthy year, 1827, and an epidemic one, 1828; and the greatest uniformity, in all these respects, existed between them; whilst, as regards easterly winds, there was a great advantage on the side of the epidemic year.

The question has often been mooted—Is yellow fever something *sui generis*, or is it but an aggravated form of remittent fever? To this, Major Tulloch declines formally to reply, though we think a very satisfactory reply is contained in the statistics of the two diseases; for whilst the former destroys at the rate of 1 in 3⅘ of those attacked, and so many are attacked, that 443 of the garrison perish in four months; the other is fatal to 1 of 11 attacked, and the admissions are so sparing, that in nineteen years only 28 deaths arise from it. The following remark tends, we would say, to confirm the conclusion deducible from statistics:

"In Gibraltar the same individual has seldom been attacked twice, even though a long series of years may have elapsed since he first suffered from it; but in the West Indies, and on the west coast of Africa, a former attack of remittent fever secures no such immunity,—indeed it could be proved, by reference to returns from these stations, that in many corps every soldier must have been treated, on the average, twice or thrice for remittent fever, during the four years of his service there." (*Ib.*, p. 9, *a.*)

By a similar argument it might be shown, that the epidemic was not an aggravated form of common continued typhus, or any other of the ordinary fevers of the garrison.

The facts presented in the Report appear to us to prove:

1. That the cause of the disease was not diffused through the general atmosphere of the district, for the troops in the different encampments in the vicinity, and who had no communication with the town, were not attacked; but was confined within the walls.

2. That this cause was not engendered by any manifest distemperature of the seasons.

3. That the disease resulting was not a mere aggravation of any disease ordinarily existing in the garrison.

The points remaining to be investigated appear to be:—Was the cause operating within the garrison contagion, or was it what may be termed a local malaria; and, in either case, how was this cause introduced or engendered? With regard to importation, Major Tulloch rejects (and on good grounds) the opinion which we remember had a short vogue whilst the epidemic was existing, that it was introduced in a Swedish ship from the Havannah. It thus appears that the main points still remain to be answered, but the Reports have certainly narrowed the ground of controversy.

B. *Cholera.* Regarding another epidemic, cholera, these Reports contain information, which is, as usual, precise and succinct. It appears that among cavalry in the United Kingdom, of 171 attacked 54 died, or 10 in 32; of troops in Gibraltar, 459 were attacked, of whom 131 were fatal, or 10 in 35; in Nova Scotia, of 210, 59 died, or 10 in 35; in Canada, in 1832, of 259, 94 died, or 10 in 28; in the same colony, in 1834, of 97, 33 died, or 10 in 29; and in Honduras, among black troops, of 62, 20 died, or 10 in 31.

Major Tulloch appends the following remark, as a commentary to these returns: "Thus, under all the modes of treatment which may have been adopted on these different occasions, the proportion of deaths to recoveries has not varied above one fourth, showing that the remedial measures hitherto employed, can have had little if any effect in counteracting the fatal character of the disease." (*British-American Report*, p. 316.)

A reasonable demur might, we think, be raised to this conclusion from such premises. The subjects of the disease are all British troops, all receive medical assistance of the best description, and the equality of the deaths may be ascribable to the equality of the skill of the practitioners employed, not to the inefficacy of the treatment. This conclusion would have been justifiable, had he found a uniformity of fatality between groups of cases skilfully treated, and others left to the care of nature. A subsequent remark, however, is more calculated to justify such a conclusion.

"In both these years (1832 and 1834), when this epidemic prevailed, the native Indians suffered from it to the same extent as the white population. At three settlements from which returns were received, about a twelfth part of the population died in 1832, and about half that proportion when it again prevailed in 1834. Although their principal remedy consisted in swallowing large quantities of charcoal mixed with lard, almost exactly the same proportion recovered as among the white inhabitants of the towns, who possessed every advantage which the aid of medical science could suggest." (*Ib.*, p. 31, *b.*)

It should not be unremarked, in discussing the question of treatment or no treatment in cholera, that one of the ingredients in this nostrum of these "stoics of the woods," the charcoal, was much esteemed by the late Dr. Jackson, as a remedy for dysentery;* and there is sufficient analogy between this disease and cholera, to render it probable that what is useful in the one disease may be of some efficacy in the other.

Major Tulloch points out the real object to be aimed at in the treatment of cholera, the early application of means, when he says:

"In all situations, and under all modes of treatment, about one in two died of the cases in the civil, and one in three of those in the military hospitals; but from the surveillance exercised over the troops, nearly half of the cases among them were noticed in the premonitory stage, and consequently could be treated with greater prospect of success than in the civil hospitals, where the great majority of patients were far advanced in the disease before they applied for medical aid." (*Ib.*, p. 31, *b.*)

The great question of the contagiousness of this epidemic the reporter does not leave untouched, but, as others have done before him, he leaves it unsettled. He describes the course of the disease along the principal channels by which the tide of emigration and commerce flowed through the country. He presents a table, displaying its appearance at successive points along the banks of the St. Lawrence, and the lakes; and both in 1832 and 1834, the accordance between the time of its outbreak and the geographical position of the infected place, in reference to the mouth of the river, is (with one exception) so perfect, as to justify his exclamation, "this singular disease may be said to have travelled with post-like regularity!" This is a strong *primâ facie* case for contagion; but after

* Sketch of Febrile Disease, p. 271.

stating that quarantine regulations were in some instances apparently effectual, and in others of little avail, and that neither the physicians nor those in constant attendance on the sick, exhibited any peculiar liability to the disease, he adds :

"Of course it is impossible, in a limited Report of this nature, to enter fully on all the facts and arguments bearing on the important and much-disputed topic of contagion; we can only say, that all which has been adduced on either side seems to fall short of absolute proof; and even those who have had the best opportunities of forming accurate opinions, by watching the progress of the disease, are forced to admit that its origin is still involved in mystery, or, at least, that the contrariety of results can only be reconciled by supposing, that under some circumstances it may be contagious, while in others it may be the reverse." (*Ib.*, p. 32, *b.*)

The increasing fatality of the disease, with advancing years, is distinctly proved in all the reports. The following numbers we collect from the table given to illustrate this fact, in the Report of Nova Scotia. Of the total strength under 18 years of age, 18 in number, none were attacked; from 18 to 25, strength 502, 1 died, or 2 per 1000; from 25 to 33, strength 829, 30 died, or 36·2 per 1000; from 33 to 40, strength 158, 14 died, or 88·6 per 1000; from 40 to 50, strength 37, 4 died, or 108 per 1000: average total, 34·7 per 1000.

"Of 293 women attached to the different corps, 87 were attacked, and 16 died, being almost exactly the same proportion as among the soldiers. Children were remarkably exempt, for of 560 in the garrison, only 16 were attacked, and 6 died. The officers, also, suffered but little; out of a strength of 60, only 4 were attacked, all of whom recovered." (*Ib.*, p. 18, *b.*)

c. *Plague*. There has been no epidemic of plague in any territory whence the materials of these reports are derived, within the period they comprise. Two, however, are mentioned by Major Tulloch, that of Malta in 1813, and that of Corfu in 1815. Both were extremely fatal; for in that of Malta, of 5600 of the natives attacked, 4486 perished, or 80 per 100; and in that of Corfu, of 700 inhabitants attacked, 630 died, or 90 per 100. These last numbers are not official; but on the authority of Dr. Gregory, in his "Practice of Physic," Major Tulloch informs us, that of 28 soldiers attacked in Corfu, only 3 recovered; whilst in Malta there were 7 recoveries in the same number. The natives of northern climates displayed, throughout the course of this epidemic, much less susceptibility to the disease than those of the south of Europe, to which circumstance Major Tulloch remarks, the exemption of the troops may perhaps be attributed. On the question of contagion, Major Tulloch expresses no precise opinion; but in relating the progress of plague in the village of Marathea, in Corfu, he says :

"As soon as its existence was officially reported, measures were adopted for cutting off all communication between the infected districts and the capital; strong cordons of troops and police were posted round each village in which the disease had made its appearance; the inhabitants were shut up in their respective residences; no communication was permitted between them, and the approach to the capital was guarded by a double line of troops, through which no one was allowed to pass from the interior, without performing fourteen days' quarantine. Owing, it is supposed, to these precautions, the disease was confined principally to the upper and lower districts of Leftimo, in which it broke out, and the capital entirely escaped its ravages." (*Mediterranean Report*, p. 38, *a.*)

Our estimate of the great value of these documents has been so strongly expressed, and the correctness of this estimate has (we hope) been so amply confirmed by copious extracts from them, that we feel no doubt of all to whom they are accessible bestowing on the Reports themselves the attention they so amply merit. We consider medical science under deep and lasting obligations to Major Tulloch, to whom we think the greatest stickler for the honour of the medical profession may freely apply the self-glorying dictum of our neighbours,—changing a single word,—*s'il n'est pas* Medecin, *il doit bien l'être.*

---

